# Downregulation of the stress-induced ligand ULBP1 following SV40 infection confers viral evasion from NK cell cytotoxicity

**DOI:** 10.18632/oncotarget.8085

**Published:** 2016-03-15

**Authors:** Yoav Bauman, Nir Drayman, Orly Ben-Nun-Shaul, Alon Vitenstein, Rachel Yamin, Yael Ophir, Ariella Oppenheim, Ofer Mandelboim

**Affiliations:** ^1^ The Lautenberg Center for General and Tumor Immunology, The BioMedical Research Institute Israel-Canada of The Faculty of Medicine (IMRIC), The Hebrew University Hadassah Medical School, Jerusalem, Israel; ^2^ Department of Hematology Hebrew University-Hadassah Medical School, Jerusalem, Israel

**Keywords:** SV40, ULBP1, immune-evasion, NK cells, NKG2D, Immunology and Microbiology Section, Immune response, Immunity

## Abstract

Polyomaviruses are a diverse family of viruses which are prevalent in the human population. However, the interactions of these viruses with the immune system are not well characterized. We have previously shown that two human polyomaviruses, JC and BK, use an identical microRNA to evade immune attack by Natural Killer (NK) cells. We showed that this viral microRNA suppresses ULBP3 expression, a stress induced ligand for the killer receptor NKG2D. Here we show that Simian Virus 40 (SV40) also evades NK cell attack through the down regulation of another stress-induced ligand of NKG2D, ULBP1. These findings indicate that NK cells play an essential role in fighting polyomavirus infections and further emphasize the importance of various members of the ULBP family in controlling polyomavirus infection.

## INTRODUCTION

NK cells which are part of the innate immune system function mainly to kill transformed and virally infected cells [1, 2]. The activity of NK cells is regulated by inhibitory and activating NK cell receptors. The activating receptor family includes, among others, the Natural Cytotoxicity Receptors (NCRs), NKG2D and CD16 [2, 3]. The activating receptors recognize ligands that are tumor associated, self-ligands, antibodies and viral proteins [4-6]. One unique group of activating ligands is the stress-induced ligands. These ligands are swiftly induced upon the cell surface following stress such as heat shock, DNA damage, tumor transformation and viral infection [7]. Eight different stress-induced ligands were identified in humans: MICA, MICB and ULBP1-6, all of which are recognized by a single NK activating receptor, NKG2D [8]. NK recognition is the initiating event of a successful immune response, first by NK cells and consequently by other immune cells. Thus, certain pathogens have developed numerous and sophisticated mechanisms to evade NK cell attack [9]. For example, the extensively studied Herpesviruses: HCMV, EBV and KSHV use both microRNA and protein based mechanisms to evade detection by NK cells [10-12].

Polyomaviruses belong to a family of small, non-enveloped, DNA tumor viruses. They encode around six early and late proteins and two microRNAs [13]. SV40 genome consists of two sets of expressed genes. The early expressed genes (early transcripts) encode for the small and large T-antigens which are required for the viral replication. The late expressed genes (late transcripts) encode for the structural proteins (VP1-3) which are necessary for viral assembly, and for the auxiliary agnoprotein [13] [14]. In recent years, new polyomaviruses have been isolated from human tissues, bringing the number of human members of the family to thirteen [15]. At least four of these viruses appear to be associated with human diseases; BK and JC viruses are known to be human pathogens since the 1970s [16] and the more recently discovered Merkel cell polyomavirus (MCV) [17] and trichodysplasia spinulosa-associated polyomavirus (TSV) [18].

Phylogenetic comparisons revealed that SV40 is evolutionary close to the human pathogens BK and JC polyomaviruses [13]. SV40 is readily propagated in the laboratory and has therefore served as a paradigm for polyomavirus infections [16, 19]. The native hosts of SV40 are old world African Green Monkeys (Chlorocebus) and perhaps other related species such as the rhesus macaque.

Only limited information is available on the interactions of polyomaviruses with the immune system. We have previously shown that two human polyomaviruses, JC and BK, use an identical microRNA to evade NK cells immune attack by downregulating the stress-induced ligand ULBP3 [22]. Here we show that the NKG2D stress-induced ligand ULBP1 is downregulated following SV40 infection, resulting in decreased NK cell killing of SV40 infected cells.

## RESULTS

### MCF7 cells are permissive for SV40 infection

**Figure 1:**
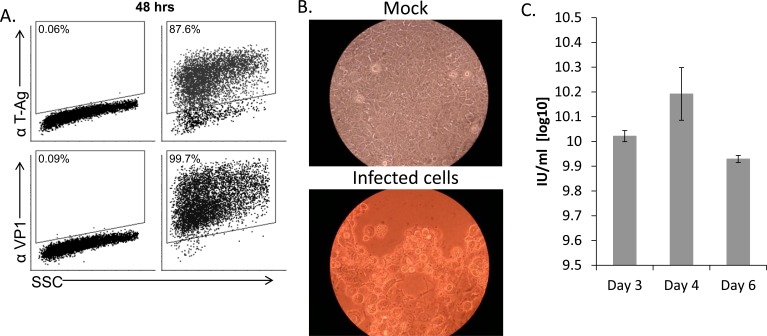
MCF7 cells support productive SV40 infection **A.** FACS staining for SV40 L-TAg (upper dot plots) and VP1 (bottom dot plots) in SV40-infected MCF7 cells (MOI 10, right two dot plots) and mock-infected cells (left two dot plots). Percentage of positive cells is indicated. **B.** Cytopathic effect as seen by light microscopy in SV40 infected cells 4-5 days post infection (MOI 10). **C.** Quantification of SV40 infectious units (IU), produced 3, 4, and 6 days post infection in MCF7 cells. Shown are mean values ± SD. No infectious units were observed in mock-infected cells. Figure shows one representative experiment out of 3 performed.

We have previously shown that the human polyomaviruses BK and JC use their microRNAs to escape NK cell attack through the down regulation of ULBP3 [22]. We therefore speculated that other polyomaviruses might also escape recognition by NK cells. We chose to focus on SV40, since this particular virus serves as a paradigm for polyomavirus infections [16, 19]. To investigate whether SV40 inhibits the NKG2D ligand expression we initially searched for a human model cell line; A cell line which supports the SV40 life cycle and is also known to endogenously express several NK ligands. As seen in Figure 1A, infection of MCF7, a human breast cancer derived cell line, by SV40 was efficient and the infected cells expressed both early (T-Ag) and late (VP1) viral proteins, at 48 hours post infection. SV40 infection of MCF7 cells results in a cytopathic effect as seen by light microscopy 4-5 days post infection (Figure 1B) and the infected cells produce infective SV40 virions (Figure 1C). Thus, we concluded that MCF7 cells support productive SV40 infection.

### The stress-induced ligand ULBP1 is downregulated following SV40 infection

**Figure 2:**
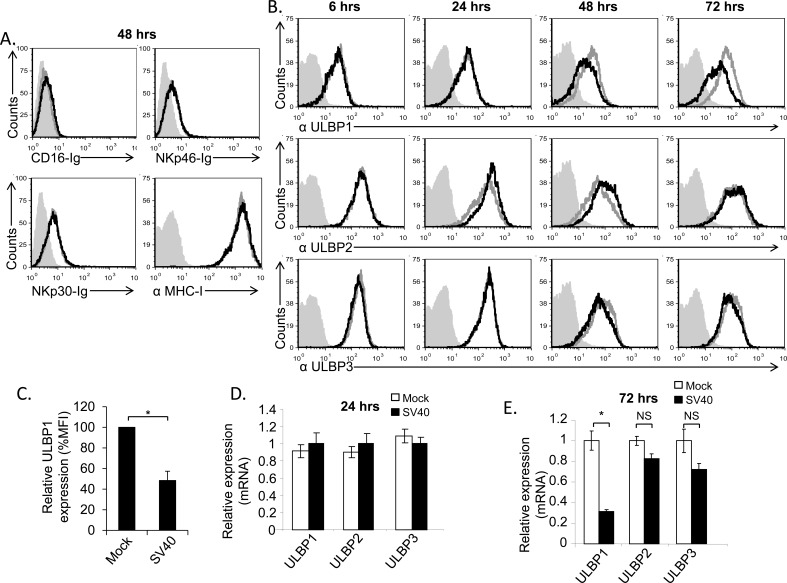
The stress-induced ligand ULBP1 is reduced following SV40 infection **A.**-**B.** FACS analysis for the expression of major NK ligands on SV40 infected MCF7 cells (black open histogram, MOI 10) compared to mock infected cells (grey open histogram). Ligands were detected by fusion proteins (A), 48 hours post infection, and by mAbs (B) at the indicated time points. The fusion proteins/mAbs used are indicated below the histograms. The filled gray histogram represents the staining of the mock cells by secondary antibodies only. The background staining of all other cells was similar to the background staining of mock cells and is not shown in the figure. Figure combines 3 independent experiments. **C.** Quantification of ULBP1 downregulation in SV40 infected cells (72 hours post infection) relative to mock cells, determined by relative MFI (Mean Florescence Intensity) reduction. Shown are mean values ± SD. Statistically significant differences are indicated (*P < 0.002, by one-tailed *t* test). Error bars (SD) are derived from three independent experiments. **D.**-**E.** Expression level of ULBPs mRNA, determined by qPCR, 24 hours (hrs) (D) and 72hrs (E) post infection, in SV40 infected cells compared to mock infected cells. Statistically significant differences are indicated (* *P* < 0.02 by one-tailed *t* test). Error bars (SD) are derived from triplicates. Results are representative of three independent experiments. NS, not significant.

The complete repertoire of the NK cells activating ligands has yet to be fully identified. In particular, the cellular ligands recognized by the NCRs are still largely uncharacterized [24, 25]. Therefore, to test whether the expression of NCR ligands is altered following SV40 infection, we made fusion proteins composed of the extra cellular portion of NKp46 and NKp30 fused to human IgG1 (named NKp46-Ig and NKp30-Ig, respectively) and stained SV40-infected and mock-infected MCF7 cells with these fusion proteins. As a control we used the fusion protein CD16-Ig (which binds the Fc portion of antibodies, [26]). As can be seen in Figure 2A, MCF7 cells express unknown ligand/s for NKp46 and NKp30 (as positive staining of mock-infected cells was detected with both NKp46-Ig and NKp30-Ig fusion proteins) yet the expression of these ligands did not change following infection (Figure 2A). MHC class I proteins serve as ligands for NK inhibitory receptors and are targeted by many viruses [27, 28]. However, no change in MHC class I proteins expression was observed during SV40 infection (Figure 2A). We previously demonstrated that ULBP3 expression was suppressed by the microRNAs of BKV and JCV [22]. We therefore tested whether the expression of ULBP3 or other members of the ULBP family is reduced by SV40. Surprisingly, we observed that while the expression of ULBP3 and ULBP2 remained unchanged during SV40 infection (Figure 2B, quantified in Supplementary Figure 1), ULBP1 expression was significantly reduced at 48 hours following SV40 infection, reaching to a maximum of around 50% reduction 72 hours post infection (Figure 2B, quantified in Figure 2C and Supplementary Figure 1).

### Mechanism of ULBP1 down regulation

To characterize the mechanisms leading to ULBP1 downregulation, we first tested, using qRT-PCR, whether ULBP1 mRNA is reduced following infection. As can be seen in Figure 2D and in agreement with the FACS results (Figure 2B), at 24 hours post infection the mRNA levels of all ULPBs tested (ULBP1, 2 and 3) were similar, irrespective of whether the cells were mock or SV40 infected. In contrast, at 72 hours post infection, the mRNA levels of ULBP1 were substantially reduced, while the ULBP2 and ULBP3 mRNA levels remained unchanged (Figure 2E). Members of the NKG2D ligands are sometimes shed from the cells surface and this may be another mechanism through which ULBP1 is lost [8]. To test this possibility we measured by ELISA, the level of ULBP1 in the supernatants of mock-infected and SV40-infected MCF7 cells. The amounts were similar (Supplementary Figure 2), indicating that SV40 infection does not lead to ULBP1 shedding.

**Figure 3:**
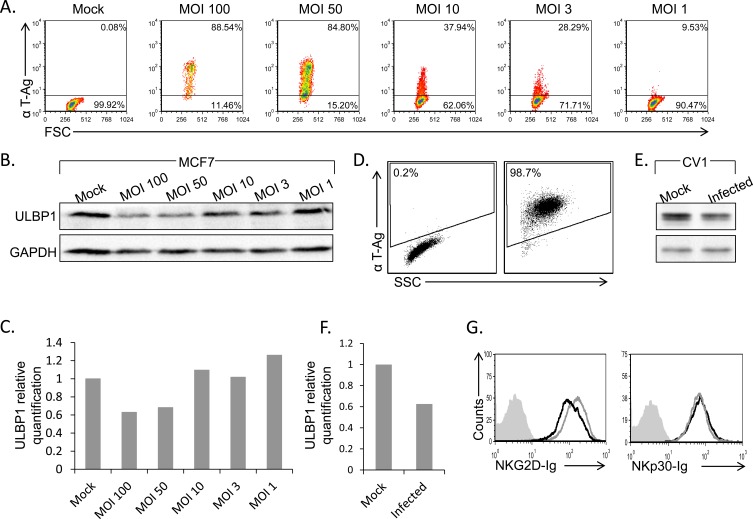
ULBP1 downregulation following infection is correlates with MOI and also observed in CV-1 cells **A.**, **D.** FACS staining for SV40 L-TAg in infected MCF7 cells at different MOIs (A, as indicated above the dot plots) or in CV-1 SV40 infected cells (MOI 10, 48h post infection, D). Percentages of positive cells are indicated. **B.**, **E.** Western blot analysis for the expression of ULBP1 (upper lanes) in SV40 infected MCF7 cells at different MOIs (B) and in SV40 infected CV-1 cells (E) compared to mock cells. GAPDH was used as control (lower lanes in B and E). Backgrounds of WB images were adjusted for better visualization. Figure shows one representative experiment out of three performed. **C.**, **F.** Relative quantification of ULBP1 expression in infected MCF7 (C) and infected CV-1 cells (F). Levels are shown relative to ULBP1 expression level in mock cells, which was set to 1. GAPDH served as normalizer in all samples. **G.** FACS analysis for the expression of NKG2D ligands or NKp30 ligands by staining of SV40 infected and uninfected CV-1 cells with NKG2D-Ig or NKp30-Ig (indicated below the histograms) (black open histogram, MOI 10), compared to mock infected cells (grey open histogram) 48h post infection.

Next, we tested whether the total levels of ULBP1 protein are reduced following infection. We infected MCF7 cells using different MOI's (MOI 1-100) which resulted in elevated rate of infection as measured by the L-Tag positive cells population (Figure 3A). Western Blot (WB) assays were then performed to determine ULBP1 level in total cell lysates. Significant, dose-dependent, down regulation of ULBP1 was observed (Figure 3B, quantified in Figure 3C).

### ULBP1 downregulation following SV40 infection is conserved

Although MCF7 cells support SV40 infection (Figure 1), human cells are not the natural host of SV40. To test whether the mechanism of ULBP1 down regulation is of biological significance, we tested whether the expression of the African Green Monkey ULBP1 protein is also suppressed following SV40 infection. Since antibodies against the monkey ULBP1 are not available, we initially tried to test for ULBP1 expression on the surface of monkey CV-1 cell line by FACS-staining, using several anti-human ULBP1 mAbs. However, none of the anti-human antibodies tested cross-reacted with the monkey ULBP1 (data not shown). In contrast, the anti-human ULBP1 mAb that worked in WB for MCF7 cells (Figure 3B) did cross-react with the monkey ULBP1. Upon infection of the green monkey CV-1 cell line with SV40 (Figure 3D), a significant downregulation of monkey ULBP1 protein was observed (Figure 3E, quantified in Figure 3F). Thus, ULBP1 downregulation during SV40 infection occurs both in human and monkey cells.

As none of the anti-human antibodies for FACS cross-reacted with the monkey ULBP1, we still wondered if we can assess the level of the NKG2D ligands on the surface of infected monkey cells. For that we have generated a human NKG2D-Ig fusion protein and stained the CV-1 cells with it. As can be seen in Figure 3G, NKG2D-Ig recognizes the monkey ULBPs. Importantly, a specific reduction in NKG2D-Ig staining of CV-1 cells was observed following SV40 infection but not that of NKp30-Ig (Figure 3G).

### The viral capsid components do not reduce ULBP1 expression

**Figure 4:**
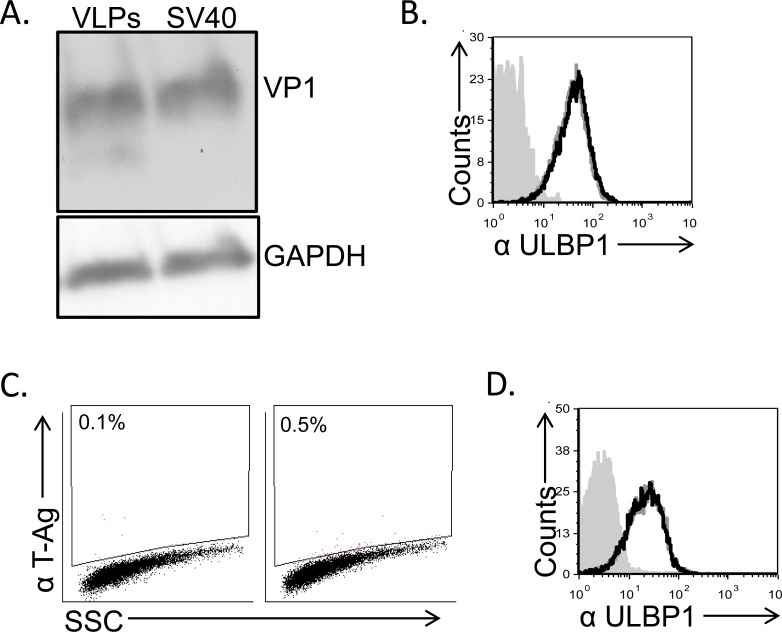
Down regulation of ULBP1 is not mediated by the viral capsid components **A.** MCF7 cells were treated with VLPs (1.2 μg) or infected with SV40 (MOI 10), extensively washed and then incubated at 37C for 6 hours. Lysates were prepared, run on SDS-PAGE gels and western blot was performed with anti-VP1 mAb. GAPDH was used as a loading control. Backgrounds of WB images were adjusted for better visualization. **B.** FACS analysis of ULBP1 expression in VLP treated MCF7 cells (black open histogram) compared to mock-treated cells (open gray histogram). The filled gray histogram represents the staining of the mock-treated cells with the secondary antibodies only. The background staining of the VLP-treated cells was similar to the mock cells and is not shown in the figure. Figure shows one representative experiment out of three performed. **C.** FACS staining for SV40 L-TAg in SV/mKate infected MCF7 cells (right dot blot) and in mock cells (left dot blot). **D.** FACS analysis of ULBP1 expression in SV/mKate infected MCF7 cells (black open histogram) compared to mock cells (open gray histogram). The filled gray histogram represents the staining of the mock cells with secondary antibodies only. The background staining of the SV/mKate-MCF7 infected cells was similar to the mock cells and is not shown in the figure.

ULBP1 downregulation could potentially be mediated by viral microRNAs, proteins or by other viral components. We could not rule out *a priori* any of the above mentioned possibilities since the down regulation of ULBP1 was observed at 48-72 hours post infection, a time point in which all viral proteins (early and late) as well as the viral microRNAs are present. Thus, to try and identify the viral component that mediates the ULBP1 down regulation, we initially examined the viral capsid. To this end, we prepared VLPs (Virus Like Particles) composed of the major capsid protein VP1 and devoid of viral DNA (Figure 4A). As can be seen in Figure 4B, MCF7 cells treated with VLPs exhibited no reduction in ULBP1 expression, indicating that ULBP1 downregulation is not mediated by the major capsid protein VP1. We next used SV/mKate (which contains all three capsid proteins VP1, VP2 and VP3), a non-replicating mutant form of SV40 virus in which the L-TAg was replaced with the mKAte gene (Figure 4C). SV/mKate infection also did not lead to reduced ULBP1 expression (Figure 4D), indicating that the SV40 capsid proteins are not responsible for ULBP1 downregulation.

### Down regulation of ULBP1 is not mediated by the viral microRNAs or the auxiliary Agno protein

**Figure 5:**
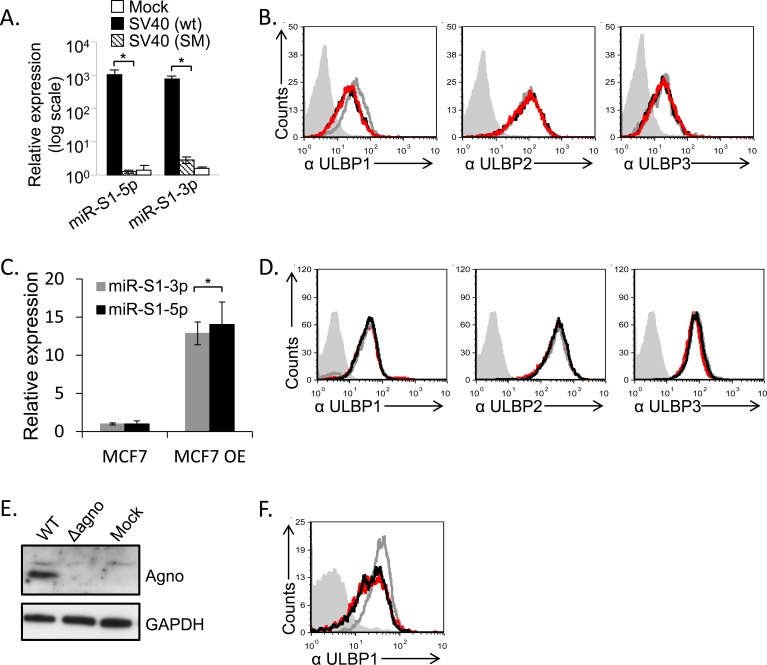
SV40 miRNAs and agnoprotein do not mediate the ULBP1 downregulation **A.**, **C.** qRT-PCR analysis for the expression of SV40 microRNAs, performed at 72 hours post infection. qRT-PCR was performed in SV40-infected and in SV40 microRNA mutant (SM)-infected MCF7 cells as well as in mock infected cells (A) and in cells transduced (designated MCF7 OE) with the viral microRNAs (C). Statistically significant differences are indicated (*P < 0.005 by one-tailed *t* test). Error bars (SD) are derived from triplicates. Results are representative of three independent experiments. **B.** FACS analysis for the expression of various ULBPs (as indicated below the histograms) at 72h following MCF7 cells infected with SV40 (black open histogram) and with SM (red open histogram) as compared to mock-infected cells (gray open histogram). The filled gray histogram represents the background staining of the mock cells by secondary mAb only. The background stainings of all other cells shown in the figure were similar to the background staining of mock cells and are not shown in the figure. Figure shows one representative experiment out of three performed. **D.** FACS analysis for the expression of various ULBPs (as indicated below the histograms), at 72h post transduction with miR-S1-5p (black open histogram) and with miR-S1-3p (red open histogram) as compared to parental cells (gray open histogram). The filled gray histogram represents the staining of the mock cells by secondary antibodies only. The background stainings of all other cells shown in the figure were similar to the background staining of mock cells and are not shown in the figure. Figure shows one representative experiment out of three performed. **E.** Western blot analysis for agnoprotein expression in SV40 (WT) and SV40 mutated in the agnoprotein start codon (Δagno), in infected MCF7 cells, or in mock infected cells (Mock). Background of WB images was adjusted for better visualization. **F.** FACS analysis for the expression of ULBP1, 72h post infection with SV40 (black open histogram) or with Δagno mutant (red open histogram) MCF7 cells compared to mock infected cells (gray open histogram). The filled gray histogram represents the mock staining of the secondary antibodies only. The backgrounds staining of all other cells shown in the figure were similar to the background staining of mock cells and are not shown in the figure. Figure shows one representative experiment out of three performed.

To test whether the SV40 microRNAs might mediate the ULBP1 downregulation we first used the SV40 SM virus (SV40 miRNA mutant) which does not express the viral microRNAs miR-S1-5p and miR-S1-3p (Figure 5A, and [23]). As seen in Figure 5B, ULBP1was still downregulated in the absence of the SV40 microRNAs. To corroborate these results we over-expressed the viral microRNAs by using lentiviral vectors. We validated that the microRNAs were indeed over-expressed (Figure 5C) and detected no change in ULBP1 expression in the presence or absence of the viral microRNAs (Figure 5D), consistent with the results obtained with the SV40 SM virus. Thus, we concluded that SV40 microRNAs do not inhibit ULBP1 expression. Since the ULBP1 reduction occurs late during infection (Figure 2B), we considered the possibility that one of the late SV40 proteins might be responsible for the ULBP1 downregulation. Since the experiments with SV40/mKate described above indicated that neither VP1 nor VP2/3 caused downregulation of ULBP1, we focused on the SV40 agnoprotein. This protein is detected late during infection, is not present in the capsid, and plays an important role in the virus life cycle [14]. We infected the MCF7 cells with the SV40 agnoprotein Pt virus that has a point mutation which prevents its expression (Figure 5E), and observed that ULBP1 expression was still reduced (Figure 5F). This indicated that the agnoprotein is not responsible for the ULBP1 down regulation.

### Ectopically expressed large T Antigen induces ULBP1 expression

**Figure 6:**
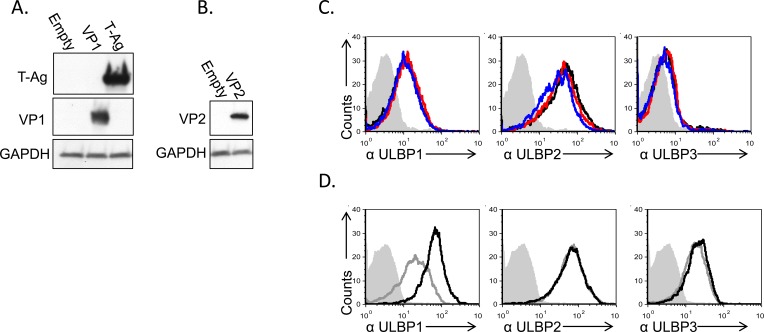
Induction of ULBP1 expression following large T-antigen expression **A.**, **B.** Western blot analysis for the expression of VP1, large T-Ag (A) and VP2 (B) in cells transduced with the relevant plasmid as indicated in the left of the figure, compared to cells transduced with empty vector. The backgrounds of WB images were adjusted for better visualization. **C.** FACS analysis for the expression of ULBPs (as indicated below the histograms) in MCF7 cells transduced with VP1 (red open histogram) or VP2 (blue open histogram) and in cells transduced with a control vector (black open histogram). The filled gray histogram represents the staining of the mock-infected cells by secondary mAb only. The background stainings of all other cells shown in the figure were similar to the background staining of mock cells and are not shown in the figure. Figure shows one representative experiment out of three performed. **D.** FACS analysis for the expression of ULBPs (as indicated below the figures) in MCF7 cells transduced with SV40 L-TAg (black open histogram), compared to cells transduced with a control vector (gray open histogram). The filled gray histogram represents the staining of the control vector cells by secondary antibodies only. The background staining of SV40 L-TAg was similar to the background staining of control vector cells and is not shown in the figure. Figure shows one representative experiment out of three performed.

At this point we excluded the involvement of several viral components in ULBP1 downregulation, including the viral microRNAs, agnoprotein and viral capsid. To further verify that the viral proteins are not involved in the ULBP1 downregulation we decided to also over express these proteins. This is because mutant viruses that do not express T-antigen, VP1, VP2 or VP3 are either not viable or less infective [29, 30]. To this end, we cloned the capsid proteins VP1, VP2/3 or the large T Antigen (L-Tag) cDNAs into lentivirus-based vectors and infected the MCF7 cells. The expression of these proteins in MCF7 cells was verified by WB (Figure 6A and 6B). The expression of VP1 and VP2/3 did not result in ULBP1 downregulation (Figure 6C). Interestingly, expression of the viral L-TAg lead to increased ULBP1 expression (around 3 folds elevation in MFI compared to control cells). Induction of ULBP1 was specific, as the expression of ULBP2 and 3 remained unchanged (Figure 6D).

### SV40 infected cells are less susceptible to NKG2D mediated NK killing

**Figure 7:**
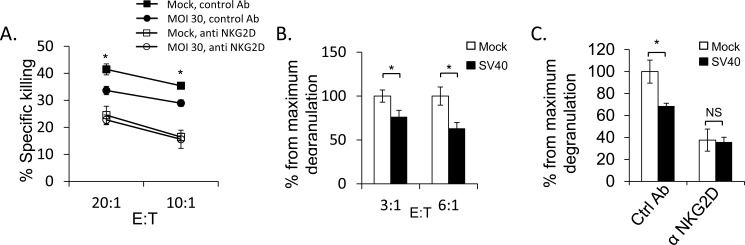
SV40 infected MCF7 cells are less susceptible to NKG2D mediated killing **A.** Bulk NK cells were pre-incubated with anti-NKG2D mAb or with isotype control mAb (control Ab). Infected or mock-infected MCF7 cells were then added and incubated for 5 hours at the indicated effector:target (E:T) ratios. Shown are mean values ± SD. Statistically significant differences are indicated (mock *versus* infected *P < 0.02, by one-tailed *t* test). Error bars (SD) are derived from triplicates. Figure show one representative experiment out of three performed. **B.** SV40 infected or mock infected MCF7 cells were incubated with bulk NK cells for 2 hours at the indicated E:T ratios and NK degranulation was determined by FACS staining for CD107a (LAMP-1) expression. The degranulation of mock infected cells was setup to be 100%. Shown are mean values ± SD. Statistically significant differences are indicated (*P < 0.005, by one-tailed *t* test). Error bars (SD) are derived from triplicates. Figure show one representative experiment out of three performed. **C.** Bulk NK cells were pre-incubated with anti-NKG2D blocking mAb or with isotype-matched control (Ctrl) mAb and then co-cultured with SV40 infected cells or mock infected cells in an E:T ratio of 3:1 for 2 hours. The degranulation of mock infected cells was setup to be 100%. Shown are mean values ± SD. Statistically significant differences are indicated (*P < 0.005, by one-tailed *t* test). Error bars (SD) are derived from triplicates. Figure show one representative experiment out of three performed.

Finally, we investigated whether ULBP1 down regulation is biologically functional. We conducted NK killing assays using primary bulk human NK cells incubated with SV40 infected MCF7 cells and mock-infected cells. A significant decrease in the killing of infected cells as compared to mock-infected cells was observed (Figure 7A). The reduction in NK killing of the infected cells resulted from reduced NKG2D recognition, as killing of all cells was equivalent when NKG2D was blocked (Figure 7A). We also performed CD107 degranulation assays which confirmed the results obtained in the NK cytotoxicity assays. As seen in Figure 7B and 7C, a significant decrease in CD107a expression on the NK cells was observed following SV40 infection. Once again, the reduction in CD107a expression was due to reduced NKG2D recognition, as CD107a expression was similar when the NKG2D was blocked (Figure 7C, raw data presented in Supplementary Figure 3).

## DISCUSSION

SV40 is the most extensively studied polyomavirus, however, its interactions with immune cells are much less investigated. Here we describe a novel immune evasion strategy employed by SV40. We show that SV40 evades NK cell killing by specifically downregulating the stress-induced ligand ULBP1 both in human and monkey cell lines. These findings, together with our previous ones [22], highlight the important role played by NK cells in fighting infections by polyomaviruses.

ULBP1 belongs to a family of proteins consisting of 8 stress-induced ligands that are recognized by NKG2D [8]. Several of these ligands are expressed on MCF7 cells. Here we showed that SV40 infection led to the specific downregulation of ULBP1, and that this downregulation was sufficient to decrease CD107a mobilization. CD107a is a marker for NK activation and degranulation, However, NK cell degranulation does not always correlate with actual killing of the target cells. Thus, we have also conducted NK cell killing which directly determines the killing of target cells and observed reduced killing of SV40 infected cells.

These results were in agreement with our previous findings which showed that even moderate reduction in one NKG2D ligand is sufficient to decrease NK activity *via* the NKG2D receptor [22]. Since changes in other NKG2D ligands were not observed following infection and since blocking of NKG2D lead to equivalent killing of all cells, we reasoned that the downregulation of ULBP1 is functionally important.

A major question in the field is why NKG2D recognizes 8 different ligands. One possible explanation is that each ligand has a different binding capacity to the NKG2D receptor [31] and this provides plasticity to the NKG2D activity when danger is sensed. Another possible explanation for the presence of multiple ligands for the NKG2D is that each of these ligands is differentially induced in response to different viruses. Indeed, we show here that ectopic expression of the viral SV40 L-Tag, resulted in specific induction of ULBP1. Thus, we speculate that the infected cells respond to the infection by sensing the L-Tag, *via* a yet unknown mechanism, by the upregulation of ULBP1. The virus, on the other hand, evades this immune response by downregulating the same protein, ULBP1. We found that the human MCF7 cell line is permissive for SV40 infection and produce infective SV40 virions, although MCF7 cells are 3-fold less susceptible to infection as compared to CV-1 (our unpublished data). The capacity of SV40 to infect humans *in-vivo* and its capacity to be transmitted horizontally between humans is highly controversial [21]. Therefore, to validate the biological significance of our findings, we also used the CV-1 cell line, derived from *Chlorocebus*, the natural host of SV40. Our results demonstrate that ULBP1 expression is also reduced during SV40 infection in CV-1 cells. Interestingly, when comparing the human sequences of ULBP1, 2 and 3 to their *Chlorocebus sabaeus* (CV-1 Chlorocebus pygerythrus related specie), ULBP1 is the most highly conserved protein among the three (90% identity for ULBP1 and around 80% for ULBP2 and 3). Thus, it is possible that SV40 which co-evolved with its natural primate host, developed mechanisms to inhibit primarily ULBP1 expression. On the other hand, BKV and JCV which co-evolved with humans, specifically inhibit the expression of ULBP3.

Using a series of mutant viruses and overexpression of viral proteins, we were unable to fully elucidate the mechanism responsible for ULBP1 downregulation by SV40. Nevertheless, we have gathered data that indicate that SV40 infection leads to an MOI-dependent reduction of ULBP1 levels, that ULBP1 is not arrested inside the cells and that the infection results in a reduction of ULBP1 mRNA levels. Furthermore, we showed that ULBP1 is not shed from the cell surface. Finally, we have ruled out the major viral components, the capsid proteins, agnoprotein and the viral microRNAs as mediators of ULBP1 downregulation. It is possible that a combination of viral components or a yet unidentified component account for ULBP1 expression reduction.

In summary, we show that SV40, similarly to other polyomaviruses (BKV and JCV), inhibits the expression of a member of the ULBP family of proteins. It will be interesting to test in the future whether additional polyomaviruses also inhibit the expression of ULBP family members as this might lead to the development of new therapies against the pathogenic members of this family.

## MATERIALS AND METHODS

### Cells, viruses and viral constructs

MCF7, a human breast cancer derived cell line (mammary gland) (ATCC HTB-22) and CV-1, an African Green Monkey, kidney derived cell line. (ATCC #CCL-70) were used throughout this study. The SM (SV40 miRNA-deficient) virus was a kind gift from Christopher S. Sullivan [23]. The SV40 agnoprotein Pt (point mutation) mutant (where the ATG codon of agnoprotein was mutated to abrogate its expression) was a kind gift from Mahmut Safak [14]. SV40 production: Wild type and SM SV40 were propagated in CV-1-PD cells, a derivative of the CV-1 cell line which have a greater capacity to support viral production [33]. Cells were harvested on the 5^th^ day post-infection by the di-detergent method [34]. Briefly, Triton X-100 and Deoxycholate were added to the culture medium to final concentrations of 1% and 0.5%, respectively. The cell suspension was centrifuged at 10,000xg for 30 min at 4°C to precipitate debris. The virus was concentrated by centrifugation at 80,000xg for 4 hr at 4°C. Viral pellet was resuspended in PBS overnight at 4°C, sonicated and centrifuged to clarify the virus suspension. For extraction of virions from SV40-infected MCF7 cells, supernatants were collected and virions were harvested by repeated freeze-thaw and chloroform treatment. To determine the Infectious virions (Infection Unit/ml) CV-1 cells were infected with supernatants obtained from the appropriate cells and the percentage of infected cells were determined by anti-Large T-antigen staining using FACS as previously described [35]. SV/mKate was produced by cloning the mKate gene (a far red fluorescent protein), instead of the T-ag. Since T-ag is required for viral propagation, this mutant was produced as above but instead of CV-1-PD cells it was grown in COS cells (ATCC CRL-1651), which supply the T-ag in trans. The virus was titrated in COS cells. mKate fluorescence was monitored for the percentage of infected cells using FACS.

VLPs production: Recombinant baculovirus expressing VP1 (Swiss-Prot P03087, PDB 1SVA) from the polyhedrin promoter were used for production of VLPs as previously described [36]. VP1 was expressed in Sf9 cells and VLPs were harvested as previously described [37]. The VLP pellet was suspended in 0.5 M NaCl, purified by ultrafiltration and stored at −20°C.

### Antibodies and fusion proteins

Anti-ULBP1, anti-ULBP2 and anti-ULBP3 antibodies were purchased from R&D systems (catalog numbers: MAB1380, 1248 and 1517 respectively) and used both for flow cytometry and for ELISA assays. Anti-ULBP1 antibody, (catalog number Sc33564, Santa Cruz Biotechnology) was used for western blotting of ULBP1. The W6/32 mAb was used for MHC-I staining. Anti-NKG2D antibody was purchased from R&D Systems (MAB139). The anti-CD99 (12E7) was used as an isotype control. Anti-CD107a (LAMP-1) was purchased from BioLegend (catalog number 328620). Anti-CD56 (Becton Dickinson) and anti-CD3 (BioLegend) antibodies were used to determine NK purity. Anti-VP2/3 and agnoprotein antibodies were produced in house as well as the rabbit polyclonal antibodies against VP1. Anti-T-Ag antibody was purchased from Abcam (Pab416). The commercial recombinant ULBP-1 Fc chimeric protein (R&D systems, catalog number 1380-UL) was used for the generation of ULBP1 standard curve.

CD16-Ig, NKp30-Ig and NKp46-Ig and NKG2D-Ig fusion proteins were generated in the human embryonic kidney 293T cells and were purified on a protein G column as described [38]. The fusion proteins used in this work were regularly assayed by SDS-PAGE protein gels, to ensure that the proteins were not degraded. Protein purity of all Ig fusion proteins used in this study was approximately 100%.

### NK cell preparation and cytotoxicity assays

NK cells were isolated from peripheral blood using the Human NK Cell Isolation Kit and the autoMACS instrument (Miltenyi Biotec) according to the manufacturer's instructions. NK cell purity was around 100% as determined by FACS. The NK cells were grown in the presence of IL-2 and the cytotoxic activity of NK cells against various targets was assessed in 5hr ^35^S release assays as described [39]. Briefly, target cells were grown over night in the presence of ^35^S- methionine added to a methionine-free media (Sigma). For each target, the spontaneous ^35^S release was measured from the supernatants of target cells which were not incubated with effector cells. Maximum ^35^S release was calculated by adding 0.1M of NaOH to the target cells. Following 5 hour incubation with effector cells, the level of ^35^S release was measured by a MicroBeta2 Plate Counter (Perkin Elmer). CD107a mobilization assays were performed as described [40]. CD107a surface expression was determined by FACS and the maximum percent of positive cells (in the mock population) was set to be 100%.

### Lentiviral constructs, production, and transduction

The SV40 L-Tag, VP1 and VP2 cDNAs were amplified from a cDNA library obtained from SV40-infected MCF7 cells. cDNAs were inserted into the pHAGE-DsRED(−)-eGFP(+) (This plasmid is a modified pHAGE-CMV-dsRed-UBC-GFP-W (Addgene), from which dsRed was excised, thus only GFP remained.) lentiviral vector which contains LTR sequences, a packaging signal, and it expresses GFP.

The following primers were used: VP1 forward: ATGAAGATGGCCCCAACAAAAA. Reverse: TCACTGCATTCTAGTTGTGGTTTGTCC. VP2 forward: ATGGGTGCTGCTTTAACACTGTT. Reverse: TTAACTCCTAGAACTTCTATTCCTCCTTT. L-TAg forward: ATGGATAAAGTTTTAAACAGAGAGG. Reverse: TTATGTTTCAGGTTCAGGGG

For the viral microRNA overexpression, artificial RNA hairpins that function as orthologues of pre-miRNA hairpins were generated by using the pTER vector [41]. Two complementary specific oligonucleotides (listed below) were annealed, phosphorylated, and inserted into the pTER vector. The artificial hairpin and H1 RNA polymerase III promoter were then excised from the vector and cloned into the lentiviral vector SIN18-pRLL-hEFIap-EGFPWRPE which contains LTR sequences, packaging signal and GFP [42]. The vector allows the simultaneous expression of both the reporter GFP and the relevant miRNA.

SV40-3p forward:

GATCCCCGCCTGTTTCATGCCCTGA GTTTCAAGAGAACTCAGGGCATG AAACAGGCTTTTTGGAAA.

Reverse:

AGCTTTTCCAAAAAGCCTGTTTCATG CCCTGAGTTCTCTTGAAACTCAGGGCATG AAACAGGCGGG.

SV40-5p forward:

GATCCCCTGAGGGGCCTGAAA TGAGCCTTTTCAAGAGAAAGGCTCATTTCAGG CCCCTCATTTTTGGAAA

Reverse:

AGCTTTTCCAAAAATGAGGGG CCTGAAATGAGCCTTTCTCTTGAAAAGGCT CATTTCAGGCCCCTCAGGG

Lentiviral vectors were produced by transient three-plasmid transfection protocol and were transfected into 293T cells using the LT1 transfection reagent (Mirus Bio LLC, Madison, WI). The transfection included the following plasmids: The pMDG envelope expression cassette (3.5ug) that expresses the VSV-G (Vesicular Stomatitis Virus Glycoprotein) envelope under the regulation of the CMV promoter [43], the gag-pol packaging construct (6.5ug) which encodes Gag, Pol, Rev, Tat, Vif, and Nef (Addgene #8455) and the relevant pHAGE-DsRED(−)-eGFP(+) plasmid (for viral protein over-expression) or SIN18-pRLL-hEFIap-EGFPWRPE (for viral microRNA over-expression) as a transfer vector (10ug). Two days after transfection, the supernatants containing viruses were collected and filtered. These viruses were then used to transduce the MCF7 cell line in the presence of polybrene (5ug/ml). Transduction efficiency was monitored by GFP levels and was around 100%.

### FACS staining

Extracellular FACS staining for ULBP 1-3 and MHC-I were performed with 0.2ug antibody per 50000 cells. The cells were washed twice and mAb binding was detected by secondary Ab (Alexa Flour^®^ 647 cat. 115-606-062). Intracellular staining of VP1 and T-ag was done as previously described [44].

### Real-time PCR

Total RNA was isolated by using Quick RNA Miniprep kit (Zymo Research) according to manufacturer instructions and RNA concentration and purity were determined. 2ug of total RNA were taken for reverse transcription with mMLV Reverse Transcriptase (Invitrogen) using Anchored Oligo-dT as the primer (Thermo Scientific). Reactions were performed for 50 min in 37°C. For SV40 microRNA detection, RNA polyadenylation was first performed on total RNA (1ug), using poly(A) polymerase (PAP) at 37°C for 1hr using the Poly(A) Tailing Kit (Ambion). For reverse transcription, 0.5ug of poly(T) adaptor (FirstChoice RLM-RACE kit; Ambion) was used according to the manufacturer's instructions. Quantitative PCR was used to determine the levels of ULBPs mRNA and for the microRNAs levels. For the detection of ULBP 1, 2 and 3 specific Taqman^®^ primers were used for amplification (Applied Biosystems). GAPDH (glyceraldehyde-3-phosphate dehydrogenase) and UBC (ubiquitin C) amplified by Specific Taqman^®^ primers (Applied Biosystems) were used as normalizers. For PCR, 50ng of cDNA were amplified with the relevant Taqman^®^ primer and Universal PCR Master Mix No AmpErase^®^ (Applied Biosystems). For SV40 microRNA expression the reaction was as follows: 2ul of cDNA was mixed with 200uM of both the forward and reverse primers and mixed with 10ul of 2× DyNAmo SYBR Green qPCR (Finnzymes). 5S rRNA and U6 snRNA were used as the endogenous reference genes for PCR quantification. The reverse primer was a 3′adaptor primer (3′RACE outer primer in the First Choice RLM-RACE kit), and the forward primer was designed based on the entire miRNA sequence. For SV40-miR-S1-5p, 5′ tgaggggcctgaaatgagcctt 3′. For SV40-miR-S1-3p, 5′ gcctgtttcatgccctgagt 3′.

### Western blotting

Cells were washed twice with PBS and total protein extracted with lysis buffer (10 mM Tris pH 7.4, 0.6% SDS). Lysates were boiled for 10 min at 99C^0^ and cell debris were pelleted by centrifuging at 14,000 rpm for 3 min. The amount of protein in each sample was determined using the Protein Assay Kit (Bio-rad). 10ug of total proteins were run on a 4-15% Mini-PROTEAN^®^ TGX™ Gel (Bio-rad) and blotted on a PVDF membrane (Millipore). Membranes were blocked in 5% skim milk in 0.05% Tween/PBS for 1 hr and incubated with primary antibodies for 1 hr at RT. Next, samples were washed 3 times in 0.05% Tween/PBS and incubated with HRP-conjugated secondary antibodies. ECL was performed and membranes exposed to films.

### ELISA

ULBP1 levels in the supernatant of SV40 infected cells were determined by ELISA. Supernatant was collected and centrifuged for 5min in 1600 rpm, and 200ul were then incubated on ELISA plates (pre-blocked with 5% FCS in PBS) for 4 hours. The plates were then washed and incubated for two hours with primary antibody (see antibodies section) and then with secondary anti mouse HRP antibody (Jackson catalog number 115035062), for 1 hour. Standard curve for ULBP1 levels was generated by using the commercial recombinant ULBP-1 Fc chimeric protein (R&D systems, catalog number 1380-UL).

### Sequences comparison

The human ULBP1, ULBP2 and ULBP3 protein sequences (uniprot accessions Q9BZM6 and Q9BZM5 and Q9BZM4 respectively) were compared to their *Chlorocebus sabaeus* predicted homologues (NCBI accessions XP_008004850.1 for ULBP1, XP_008004855.1 for ULBP2 and XP_008005740.1 for ULBP3) by using uniprot alignment http://www.uniprot.org/align/.

## SUPPLEMENTARY MATERIAL FIGURES


