# Supplementary material for: Downregulation of the stress-induced ligand ULBP1 following SV40 infection confers viral evasion from NK cell cytotoxicity
Source: Oncotarget. 2016 Mar 15;7(13):15369–81. doi: 10.18632/oncotarget.8085 (PMC4941247; doi:10.18632/oncotarget.8085)
Supplement: Supplementary file 1 [file oncotarget-07-15369-s001.pdf]

Downregulation of the stress-induced ligand ULBP1 following SV40 infection confers viral evasion from NK cell cytotoxicity

Supplementary Material

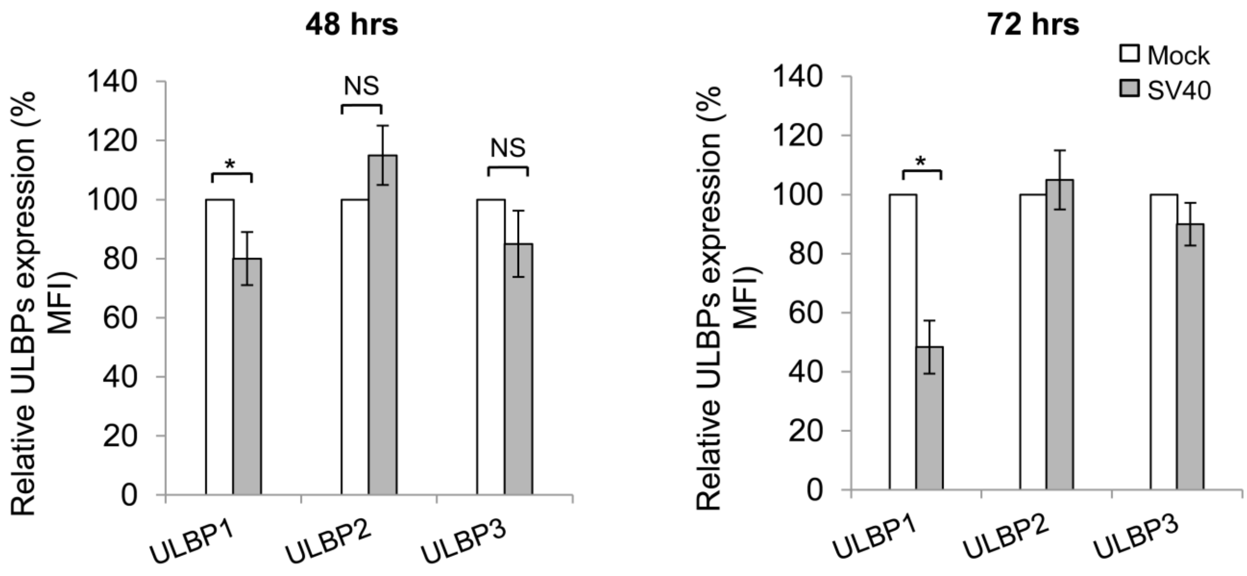

Supplementary Figure 1: Quantification of the ULBPs expression in SV40 infected MCF7 cells
Quantification of ULBPs proteins (ULBP 1-3) in SV40 infected MCF7 cells (48 and 72 hours post infection) relative to mock cells, determined by relative MFI (Mean Florescence Intensity) reduction. Shown are mean values ± SD. Statistically significant differences are indicated (*P <0.002, by one-tailed t test). Error bars (SD) are derived from three independent experiments.

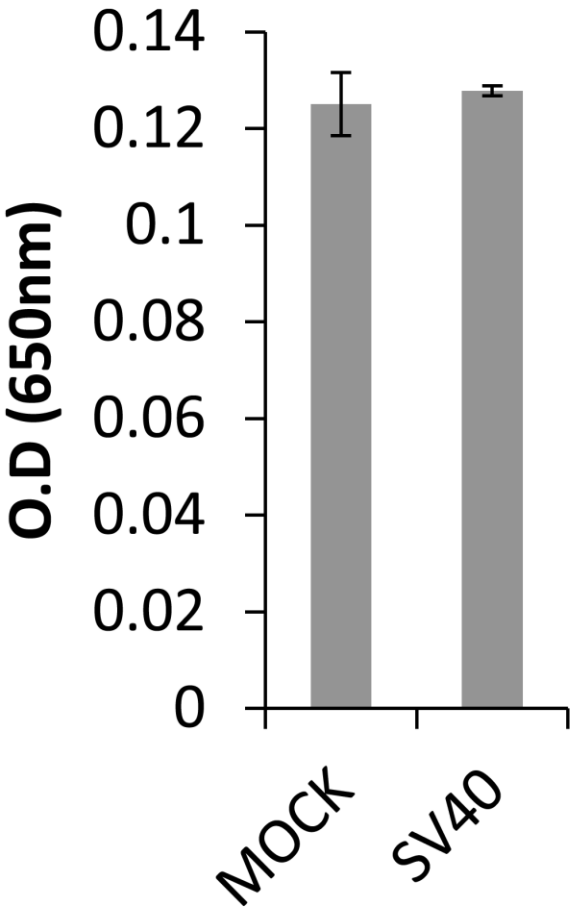

Supplementary Figure 2: Enhanced cleavage of ULBP1 is not seen following SV40 infection
Soluble ULBP1 protein levels as measured by ELISA (O.D 650nm) found in the supernatants of MCF7 cells infected with SV40 (right column) as compared to Mock cells (left column) 72 hours post infection.

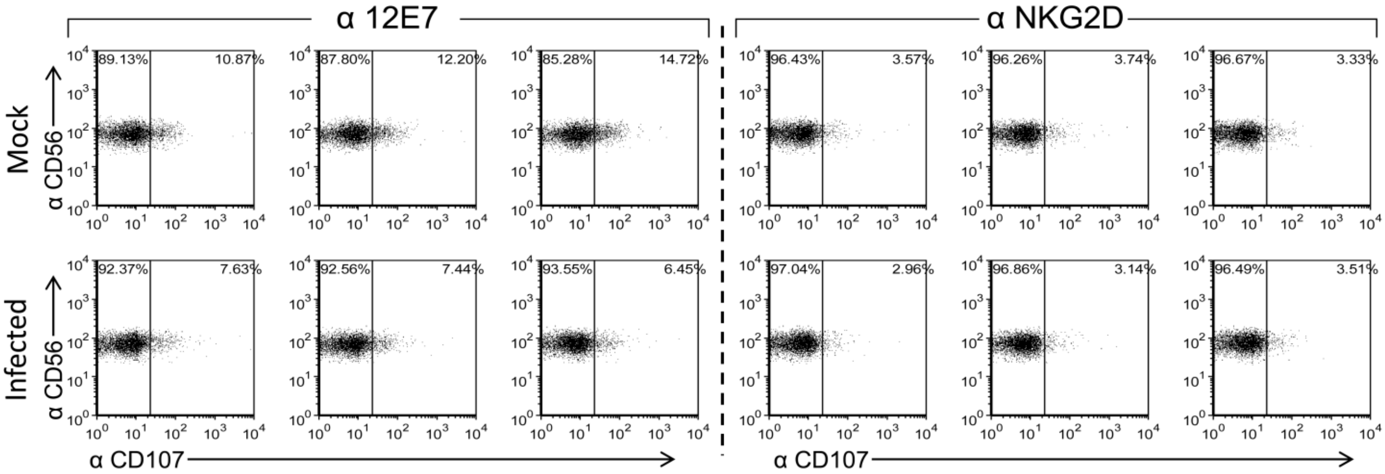

Supplementary Figure 3. The reduction in NK cells degranulation following SV40 infection is NKG2D-dependent
FACS analysis for the expression of CD107a (x-axis) and for CD56 (y-axis) on NK cells pre-incubated with anti-NKG2D blocking mAb or with isotype-matched control mAb (12E7). NK cells were co-cultured with SV40 infected cells (designated infected) or with mock infected cells (designated Mock). Percentage of CD107a positive cells is indicated in the dot blots. The figure shows the row data of figure 7C.
